# Malicious Mushrooms

**DOI:** 10.1016/j.gastha.2023.01.012

**Published:** 2023-01-20

**Authors:** Peter Iskander, Diana Marzouk, Anthony Iskander, Fouzia Oza

**Affiliations:** 1 The Wright Center for GME, Scranton, Pennsylvania; 2 Virginia Commonwealth University, Richmond, Virginia; 3 Xavier University School of Medicine Aruba, Oranjestad, Aruba

**Keywords:** *Amanita phalloides*, Silibinin hemisuccinate, N-Acetylcysteine, Acute Liver Injury, Liver Transplant

## Abstract

Accidental ingestion of the toxic Death Cap mushroom, and others of the *Amantina* species, can occur due to their physical similarities with commonly edible fungi. Production of certain toxins which prevent protein synthesis can lead to fulminant organ failure and death. Although treatment is mostly supportive due to a lack of specific antidote, early recognition can aid in meaningful recovery. Nonspecific symptoms are generally present early in the course and, therefore, high index of suspicion is required. We present 2 cases of suspected *Amanita phalloides* poisoning leading to acute liver injury; one leading to resolvement of symptoms and the other being fatal.

## Introduction

*Amanita phalloides*, also commonly known as the Death Cap, is one fungus that is genuinely true to its name. It is known as one of the most lethal mushrooms in the world; it has been the cause of many human fatalities, likely due to the fact that it closely resembles a few other edible mushrooms such as the Caesar or Straw mushroom.^1^ In terms of deaths due to mushroom ingestions, up to 95% can be attributed to the amatoxin produced by these fungi; fulminant liver failure is a severe complication that can result.^2^ Its main mechanism of toxicity is via amatoxin which inhibits protein synthesis leading to cell death. Initially, its effect can be seen as nonspecific gastrointestinal symptoms which can progress to fulminant liver failure in a few days. This is likely due to the fact that it is rapidly absorbed into the portal system where it, in turn, is transported to the liver. As there is no official antidote, mainstay of therapy is symptomatic and conservative; controlling fevers and replacement of electrolytes, fluids, and clotting factors.

## Case Report

Two patients presenting to the hospital after ingesting mushrooms; per their description, high suspicion was made for *Amanita phalloides* (Death Cap) poisoning.

### Case 1

A 38-year-old male admitted to the hospital with severe abdominal pain. Found to have elevated liver enzymes with AST 2123, ALP 2402, ALP 76, T bili 2.5. He was also found to be oliguric, has high ammonia levels, and an INR peak of 10. He was treated with an antitoxin IV Silibinin hemisuccinate and N-acetyl cysteine. He began developing an acute kidney injury for which he was treated with aggressive fluid resuscitation. After a few days of management, liver enzymes began to downtrend and the patient was discharged in a stable condition.

### Case 2

A 51-year-old male with no significant past medical history who presented to the hospital with severe abdominal pain associated with nausea and vomiting after ingesting mushrooms; suspicion was made for *Amanita phalloides* (Death Cap) poisoning. Laboratories on admission significant for AST 3860, ALT 2710, T bili 4.1 INR 2.3, Ammonia 133, MELD-Na 23. He was started on N-acetyl cysteine and IV Silibinin hemisuccinate. He progressively started to become more confused and began developing seizure-like activity for which he was admitted to the intensive care unit. Magnetic resonance imaging of the head revealed cerebral edema and, ultimately, plan was made to intubate and sedate the patient as there was further concern of him no longer being able to protect his airway. Patient’s clinical status progressively continued to deteriorate. He began showing signs of hemodynamic shock and lactic acidosis for which he required vasopressor support and stress dose steroids. CRRT and IV Sodium Bicarbonate were initiated as his kidney function and urine output continued to decline. Despite transfusions, patient continued to become coagulopathic with hemoglobin/hematocrit and platelets dropping to as low as 5.5/16.9 and 16, respectively. Despite all aggressive measures, decision was made to transition patient to comfort care after 11 days of admission; patient expired peacefully later on that day.

## Discussion

### Pathophysiology

*Amanita phalloides*’s deadly nature can be attributed to its 3 main groups of toxins: amatoxins, phallotoxins, and virotoxins.^3^

Amatoxin is a heat-stable toxin, meaning that regardless of whether or not the mushroom is ingested raw or cooked, its poisonous characteristics persist. It is readily picked up into the portal system from the gastrointestinal tract which may explain its heavy burden on hepatic toxicity. It then binds to RNA Polymerase II, causing an overall inhibition, leading to an arrest of protein synthesis. Ultimately, this leads to cell death. Up to half of those who progress to amatoxin-induced liver failure will require referral for liver transplant evaluation.^4^ Amatoxin has a lethal dose of roughly 0.1–0.4 mg/kg which is concerning as a single mushroom can contain up to 15 mg.^5^

Phallotoxins and virotoxins work similarly in that they both have a particular affinity to hepatocytes. They work by inhibiting microfilament depolymerization. This can lead to a decrease in bile acid flow resulting in cholestasis.^6^ Although they can be harmful, they do not play a specific part in the poisonous effects of *Amanita*.

### Complications

Nausea and vomiting are the most common side and acute effects in those who have ingested mushrooms of the *Amanita* species. As the toxin’s effects spread coagulopathy, encephalopathy, liver/kidney failure, and intracerebral hemorrhage and edema can develop.^7^ Intoxication can be explained in 4 phases. In phase 1, the lag phase, patients can remain asymptomatic for roughly 0–6 hours. Phase 2 occurs within 6–24 hours which can include gastroenteritis-type symptoms: nausea, vomiting, abdominal cramping, and severe diarrhea. This is significant as it can lead to severe diarrhea and hemodynamic shock. Phase 3, within 24–36 hours, is where patients can seem to symptomatically improve despite a continuous rise in liver enzymes; this is termed the latent phase. The fourth phase, usually occurring after 36 hours, is the time when considerable liver injury can arise. This can lead to coagulopathy, jaundice, encephalopathy, coma, seizures, and death.^8^

### Treatment

As there is no specific acute antidote to these toxins, main treatment is focused on supportive management until the toxin is able to be cleared. This includes aggressive fluid replacement secondary to gastrointestinal losses, electrolyte replacement, replacement of clotting factors, etc.

N-acetylcysteine has been used as a form of therapy for those showing signs of acute liver injuries. Its mainstay of action is acting as an antioxidant picking up free radicals.^9^ It is generally safe and well tolerated among many patients.

Penicillin G has been used in the past as part of a therapeutic regimen; per a 20-year retrospective analysis by Enjalbert et al in 2002, it was shown to have little to no benefit as a solo therapy. Its inclusion in a multitherapy regimen with N-acetylcysteine and silybylin proved to be more effective, however.^10^

Silibinin is an extract from seeds of the Milk Thistle plant. Its key to fame is its ability to stimulate liver regeneration by increasing the expression of the 5.8s, 18s, and 28s ribosomal RNA by up to 20%.^11^ This leads to a stimulation of protein transcription and synthesis.^12^ This, in theory, can therefore help replete the hepatic cells damaged by the amatoxin and slow down the progression to liver failure.

Activated charcoal and other remedies have also been used, but overall, in patients who show evidence of liver failure, their condition usually proves fatal within 1–2 weeks. It is at these times where liver transplant usually becomes the last resort for survival.^13^

Mushroom poisoning can vary significantly in terms of symptoms from nonspecific gastrointestinal upset to fulminant fatal organ failure. Current management and treatment can be just as variable. At this time, the mainstay of treatment is supportive management but, ultimately, those who develop severe organ damage (mainly liver) may require transplant. Good education may be the first step in preventing adverse outcomes. As these toxic mushrooms resemble more commonly edible ones, those who forage or pick fungi from nature may need to better familiarize themselves with certain characteristics that can differentiate them from the safer ones; this can literally be the difference between life and death. Even per Centers for Disease Control and Prevention guidelines, “Inexperienced foragers should be strongly discouraged from eating any wild mushrooms.”^14^ Recognition remains difficult in the acute setting due to the toxins' delayed onset of symptoms and so a high degree of suspicion should remain on the differentials to help prevent long-term complications.^15^
